# Characterization of the second- and third-harmonic optical susceptibilities of atomically thin tungsten diselenide

**DOI:** 10.1038/s41598-018-28374-1

**Published:** 2018-07-03

**Authors:** Henrique G. Rosa, Yi Wei Ho, Ivan Verzhbitskiy, Manuel J. F. L. Rodrigues, Takashi Taniguchi, Kenji Watanabe, Goki Eda, Vitor M. Pereira, José C. V. Gomes

**Affiliations:** 1 Centre for Advanced 2D Materials (CA2DM), National University of Singapore, 6 Science Drive 2, Singapore, 117546 Singapore; 2 NUS Graduate School for Integrative Sciences and Engineering (NGS), Centre for Life Sciences (CeLS), 28 Medical Drive, Singapore, 117456 Singapore; 3 Department of Physics, National University of Singapore, 2 Science Drive 3, Singapore, 117551 Singapore; 4 Center of Physics and Department of Physics, Universidade do Minho, 4710-057 Braga, Portugal; 5 National Institute for Materials Science, 1-1 Namiki, Tsukuba, 305-0044 Japan; 6 Department of Chemistry, National University of Singapore, 3 Science Drive 3, Singapore, 117543 Singapore

## Abstract

We report the first detailed characterization of the sheet third-harmonic optical susceptibility, *χ*^(3)^_s_, of tungsten diselenide (WSe_2_). With a home-built multiphoton microscope setup developed to study harmonics generation, we map the second and third-harmonic intensities as a function of position in the sample, pump power and polarization angle, for single- and few-layers flakes of WSe_2_. We register a value of |*χ*^(3)^_s_| ≈ 0.9 × 10^−28^ m^3^ V^−2^ at a fundamental excitation frequency of *ℏω* = 0.8 eV, which is comparable in magnitude to the third-harmonic susceptibility of other group-VI transition metal dichalcogenides. The simultaneously recorded sheet second-harmonic susceptibility is found to be |*χ*^(2)^_s_| ≈ 0.7 × 10^−19^ m^2^ V^−1^ in very good agreement on the order of magnitude with recent reports for WSe_2_, which asserts the robustness of our values for |*χ*^(3)^_s_|.

## Introduction

Atomically thin two-dimensional crystals of semiconducting transition-metal dichalcogenides (TMD) are currently a subject of intense research. The most prominent members of this family have been molybdenum disulfide (MoS_2_) and diselenide (MoSe_2_), as well as tungsten disulfide (WS_2_) and diselenide (WSe_2_). Their unique electronic, optical and structural characteristics are under active scrutiny for applications in photonics, optoelectronics and electronic devices. In addition to the ability of tuning their carrier densities on demand by field-effect, such properties include high charge-carrier mobility^1,2^, a direct band gap in monolayer crystals that evolves to indirect with additional layers^3^, photoluminescent emission in the visible-NIR spectral range^4–6^, high nonlinear optical susceptibilities^7,8^, strong spin-orbit coupling^9,10^ and spin-valley locking and novel valleytronics phenomena^11^.

Owing to their semiconducting character and favorable band gaps for conventional optoelectronics^12^, the pressing need for detailed characterization of their intrinsic optical response has stimulated a steady and broad array of experimental results^12,13^. In particular, nonlinear optical applications hinge upon the nature of the second and third-order optical susceptibilities (*χ*^(2)^ and *χ*^(3)^_,_ respectively), which are conventionally obtained from harmonic generation experiments. Whereas second-harmonic generation (SHG) in the most prominent TMD had been studied in a number of recent experiments^7,8,14–16^, reports on third-harmonic generation (THG) among this family have been scarce^17,18^. Remarkably, THG in either single or few-layer selenide-TMD remains unexplored. These results on harmonic generation in TMD and in other 2D layered materials have been summarized in a recent review by Autere *et al*.^19,20^.

In this context, we have used multiphoton spectroscopy to simultaneously measure SHG and THG intensities of exfoliated single- and few-layers WSe_2_. By studying the harmonics generation as a function of pump power, layer-dependence and spatial position, and correlating the data with atomic force microscopy, we are able to report a robust characterization of third-harmonic *χ*^(3)^. We demonstrate that THG is independent on the polarization angle and is proportional to the square of the number of layers. We further show that the magnitude of WSe_2_ *χ*^(3)^ is comparable to that of sulfides of the same TMD family. Even though this type of experiment has been broadly applied to determine *χ*^(2)^ and *χ*^(3)^ in several two-dimensional materials^14,18,21^, the characterization of THG and a quantitative measurement of *χ*^(3)^ in WSe_2_ had remained unknown.

## Results and Discussion

**Figure 1:**
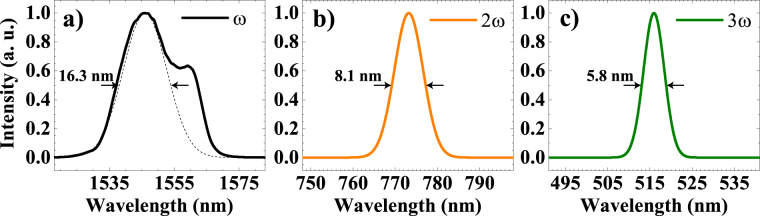
Spectra for (**a**) 1550 nm pump, (**b**) SHG at λ_central_ = 773 nm and (**c**) THG at λ_central_ = 516 nm. The pump beam full-width at half-maximum (FWHM) bandwidth is 25.1 nm, but only the peak centered at 1546 nm contributes for the harmonic generation (dotted line, FWHM bandwidth ~ 16.3 nm). The FWHM bandwidth and central wavelength for SHG are 8.1 nm and 773 nm, and for THG are 5.8 nm and 516 nm, respectively.

We investigated the nonlinear optical properties of mechanically exfoliated atomically thin 2H-WSe_2_ (2H polytype) with a home-built multiphoton microscope specifically designed for harmonics generation. Before nonlinear optical experiments, the sample was extensively characterized by photoluminescence and Raman spectroscopy, as well as by atomic force microscopy (for details on the sample preparation, characterization and experimental setup, see materials and methods section and supporting information). The pump wavelength for the harmonic experiments is 1546 nm, therefore SHG emission is centered at 773 nm and THG emission at 516 nm, as shown in Fig. 1.

**Figure 2:**
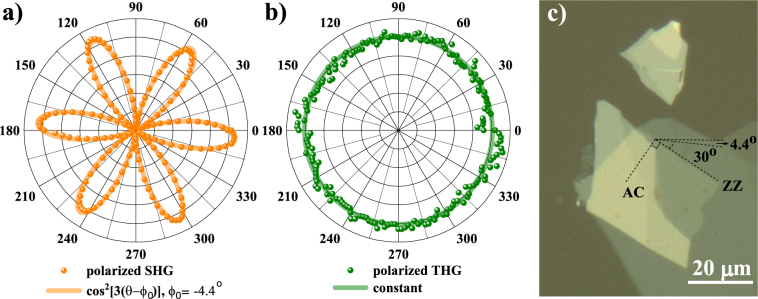
Polarized harmonic generation in WSe_2_: (**a**) SHG, *ϕ*_0_ = −4.4° and (**b**) THG, constant intensity with *θ*; (**c**) optical image of the sample with armchair (AC) and zig-zag (ZZ) directions.

The absolute orientation of the WSe_2_ crystal, relatively to the laboratory frame, was determined by polarized second-harmonic generation (pSHG), where the incident pump linear polarization and a parallel polarizer were rotated simultaneously by the same angle *θ* while recording the spectrum. Since 2H-WSe_2_ belongs to the D_3h_ point group^22^, as expected from the threefold rotational symmetry along the *c* crystallographic axis and demonstrated for a number of odd-layered TMD of this family, the pSHG intensity is proportional to cos^2^[3(*θ*-*ϕ*_0_)], where *ϕ*_0_ is the angle of the armchair direction of the flake in the laboratory frame. The result of our pSHG is shown in Fig. 2a. This strong polarization dependence makes pSHG a preferred tool for fast crystallographic alignment of these materials^7,8,14,23^. In contrast, the THG is polarization independent, as can be seen from Fig. 2b, as expected for all other crystals belonging to the D_3h_ group^18^. Figure 2c shows an optical image of the sample with the identification of armchair (AC) and zig-zag (ZZ) directions of the flakes^8^.

We hence determined the AC direction to lie at *ϕ*_0_ = − 4.4° + m × 60°, m∈Z, and set the pump polarization parallel to this direction of maximum response for our subsequent SHG and THG experiments.

**Figure 3:**
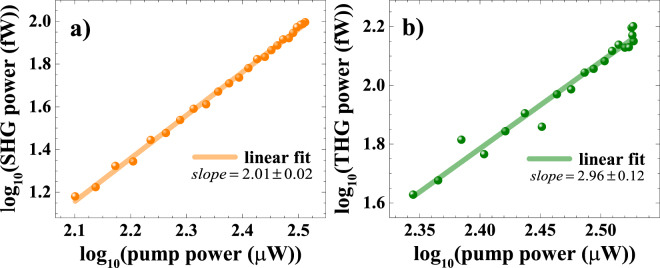
Double logarithmic plots of the power-dependent nonlinear signals for (**a**) the SHG of single-layer and (**b**) the THG of 9-layers WSe_2_.

To assert the second and third-harmonic nature of the signals discussed so far, we measured their dependence on the pump power via Malus’ law experiment, by inserting a fixed polarizer before the sample in the experimental setup used for pSHG measurements (for more details on the experimental setup, see supporting information). Additionally, we carried out a calibration leading to actual average power readings from spectrometer counts in order to extract the magnitude of the susceptibilities (for more details on calibration factors, see supporting information). Figure 3 shows power-dependent double logarithmic plots of SHG and THG actual average power. The power scaling of the SHG and THG intensities is shown to follow very well the respectively expected quadratic and cubic dependences.

**Figure 4:**
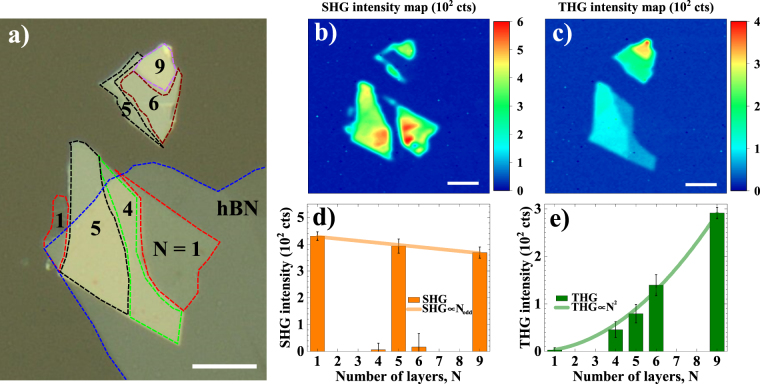
(**a**) Optical image of the sample with labeled number of layers (*N*); (**b**) Spatial SHG and (**c**) THG intensity mappings across the WSe_2_ sample; (**d**) Spatial average SHG and (**e**) THG intensities as a function of *N*. The values and error bars indicated for the SHG and THG correspond to the mean ± 3 × (standard deviation). Scale bars = 10 µm.

To acquire spatial maps, the sample was raster scanned across the pump beam in 0.5 µm steps, while SHG and THG intensities were recorded simultaneously. The results are shown in Fig. 4. An image of the sample with the number of layers (*N*) labeled in each region, is shown in Fig. 4a. The thickness assignment was performed by correlating atomic force microscopy data with the SHG contrast. The image also shows the presence of a thin film of hexagonal boron nitride (hBN) partially covering the WSe_2_ multilayer, which was used to prevent environmental degradation (for more details on sample fabrication, see supporting information).

Figure 4b shows the sharp SHG contrast between regions of odd- and even-*N*, as SHG in these TMD is expected for odd-*N* flakes^24,25^, with a decreasing intensity for increasing number of layers^10,26^. Yet, even though SHG is expected to be strictly zero for even-*N*, a residual non-zero intensity is recorded for 4- and 6-layers regions, as shown in Fig. 4d. This effect might be attributed to incomplete destructive interference between SHG from adjacent layers, as discussed previously by Li *et al*.^25^, although its fundamental cause remains unexplored. Moreover, this effect might be responsible for a small negative slope for the SHG intensity as a function of the odd-*N*. Extrapolating the linearly decreasing trend inferred from Fig. 4d, significant SHG intensity in WSe_2_ should be observed for samples with up to ~50 layers. However, the challenges in unambiguously determining *N* for samples with more than 10 layers make this observation difficult.

Figure 4c shows the THG map of the sample. In addition to the signal now arising from all regions of the sample, the key observation is that the intensity clearly scales up with the number of layers. To be more specific and quantitative, we compiled the average intensity over regions with same *N* in Fig. 4c. The THG intensity is proportional to *N*^2^, as shown in Fig. 4e. This quadratic scaling with thickness is a direct evidence that we can consider each layer as contributing independently to the overall THG, since the linear relation of the third-harmonic susceptibility $|{{{\chi }}^{(3)}}_{{N}}|={N}\times |{{{\chi }}^{(3)}}_{s}|$ indeed implies in ${{I}}_{{\rm{THG}}}\propto |{{{\chi }}^{(3)}}_{{N}}{|}^{2}\propto {{N}}^{2}\times |{{{\chi }}^{(3)}}_{s}{|}^{2}$.

No border effect for enhancement of SHG or THG was observed. In fact, DFT calculations for chemical vapour deposited (CVD) WS2 showed that at the borders the bandgap becomes indirect^27,28^, which actually contributes to quenching of SHG and, probably, THG at the edges.

A direct comparison between Fig. 4a and c indicates that regions with same *N* have slightly weaker harmonic intensities when covered by hBN, which the most notable case being the 5-layers region. This can be explained by the Fresnel reflection/transmission coefficients, since the total transmittance depends on the layer stacking, total sample thickness and refractive index mismatch. We observed that, by recording the transmitted power at the fundamental frequency with a reference photodetector, regions without hBN may have up to 4% higher pump transmittance than covered regions (for more details on reference transmittance mapping, see supporting information). This difference in pump power leads to SHG and THG intensities of, respectively, 8% and 12% higher in non-covered regions. The analysis presented in Fig. 4d and e already corrects the intensities by the pump power in each region. The contribution to SHG and THG from hBN solely is negligible, as we have confirmed by pumping hBN regions of the sample (Fig. 4b and c). This agrees with previous report by Li *et al*.^25^, where the authors show that hBN |*χ*^*(2*)^_s_| is 2 orders of magnitude lower than that of TMD.

Taking advantage of the spatial resolution and well defined layer assignment in our experiment, the magnitude of the nonlinear susceptibilities *χ*^*(2*)^ and *χ*^*(3*)^ for WSe_2_ were extracted from the harmonic mapping by using the model deducted by Woodward *et al*.^18^. Simple modifications were implemented in the model, accounting for harmonic generation in transmittance, with fundamental beam pumping the sample from the substrate-sample interface, as shown in Equation (1) and (2):

1 $$|{{\chi }^{(2)}}_{{\rm{s}}}|=\sqrt{\frac{P(2\omega )}{{P}^{2}(\omega )}\times \frac{c.{\varepsilon }_{0}.RR.A.{\rm{\Delta }}\tau .{\lambda }^{2}}{64\sqrt{2}{\pi }^{2}S}\times \frac{{(1+n)}^{6}}{{n}^{3}}},$$

2 $$|{{\chi }^{(3)}}_{{\rm{s}}}|=\sqrt{\frac{P(3\omega )}{{P}^{3}(\omega )}\times \frac{{c}^{2}.{\varepsilon }_{0}^{2}.R{R}^{2}.{A}^{2}.{\rm{\Delta }}{\tau }^{2}.{\lambda }^{2}}{336\sqrt{3}{\pi }^{2}{S}^{2}}\times \frac{{(1+n)}^{8}}{{n}^{4}},}$$

where *P(2ω)* and *P(3ω)* are second-harmonic and third-harmonic average powers, respectively, and *P(ω)* is the fundamental average power. *c* is the speed of light in vacuum, *ε*_0_ is the vacuum electric permittivity. *RR* is the repetition rate (80 MHz), *A* is the minimum spot area (2.0 ± 0.3 μm^2^, obtained from the half-width at 1/e^2^ beam radius of 0.8 ± 0.1 µm), *Δτ* is the full-width at half maximum pulse duration at the sample spot (200 ± 10 fs) and *λ* is the wavelength (1545 nm) for the fundamental beam. Finally, *n* is the substrate refractive index at the fundamental wavelength (1.47 at 1545 nm) and S = 0.94 is a shape factor assuming Gaussian pulses. The average power of the fundamental beam was kept constant at 0.65 mW, and the calibration factors to obtain harmonic average powers from counts, as in Fig. 4, are 0.275 fW counts^−1^ and 0.512 fW counts^−1^, respectively, for SHG and THG. |*χ*^(2)^| and |*χ*^(3)^| results are summarized in Table 1.

**Table 1:** Harmonic susceptibilities of WSe_2_ as a function of number of layers *N*.

Number of layers (*N*)	|*χ*^(2)^_N_| (10^−19^ m^2^ V^−1^)	|*χ*^(3)^_N_| (10^−28^ m^3^ V^−2^)
1	0.70 ± 0.09	0.9 ± 0.2
4	0.08 ± 0.08	3.4 ± 0.5
5	0.67 ± 0.09	4.5 ± 0.6
6	0.2 ± 0.1	6.0 ± 0.8
9	0.65 ± 0.08	8.6 ± 0.9
Average/sheet (per layer)	—	0.91 ± 0.07
	|*χ*^(2)^_b_| (10^−10^ m V^−1^)	|*χ*^(3)^_b_| (10^−19^ m^2^ V^−2^)
Bulk susceptibility	0.9 ± 0.1	1.16 ± 0.09

The reported values correspond to the mean ± standard deviation. The error values are obtained from the uncertainties of the relevant experimental parameters present in equations (1) and (2), through classical error propagation theory.

As only odd-*N* regions contribute with appreciable SHG, the second-order susceptibility is expressed in terms of an effective value |*χ*^(2)^_N_|, which was directly obtained from each region on the WSe_2_ sample. The third-order susceptibility is expressed in terms of both an effective value, |*χ*^(3)^_N_|, and the average per layer, |*χ*^(3)^_s_|. The bulk-like values are obtained from the expression |*χ*_b_| = |*χ*_s_|/*δ*, where *δ* is the inter-layer distance of the flake (for our WSe_2_ flake, *δ* = 0.79 ± 0.02 nm – for more details on the thickness characterization, see supporting information).

The values obtained for sheet and bulk second-order susceptibility agree with those previously reported^7,29^ within the order of magnitude, and the sheet and bulk values of third-harmonic susceptibility are reported here for the first time. The *χ*^(3)^ magnitude is comparable to other TMD^18,30^ and is larger than that reported for graphene^18,31^. Although the presented results have small errors inherent from the model and parameters we used (~13% for SHG at odd-*N*, ~13% for THG – except *N* = 1 with 20% error), those should only be taken as a good estimative for the order of magnitude of such susceptibilities, since their determination relies on the precise measurement of many important parameters (which can vary upon definitions and measurement techniques). The measurement of harmonic susceptibilities of two-dimensional layered materials is known to be sensitive to the fabrication process (Woodward *et al*.^18^ reported 26% variation between exfoliated and CVD MoS_2_), stacking order, and surrounding environment (substrate and superstrate)^19^.

Excitons are strongly present in semiconductor TMD, excitonic effects may also play role by enhancing nonlinear optical transitions, as reported previously for MoS_2_^8^ and for WSe_2_^32,33^: when the harmonic photon energies are in resonances to excitonic or single-particle energy levels from the material, harmonic signals up to 1 order of magnitude higher might be observed. Since in our experiment WSe_2_ is pumped by photons of 0.8 eV, no resonant enhancement effect is expected to take place, because both SHG (1.6 eV) and THG (2.4 eV) are off-resonance with energy levels of the material^34^. Nevertheless, our SHG results agrees to those off-resonance published results. Although no resonance has been observed, the presented characterization provides important parameters to support the realization of 2D-materials-based devices for applications in telecommunication systems and silicon photonics.

Benefiting from the small sample thickness and THG efficiency, multi-layer WSe_2_ appears to be a potential material for nonlinear optical applications as, for example, on-chip optical frequency conversion^35^, silicon photonics^36,37^, or other third-order related phenomena like all-optical switching^38,39^, which depends on a different *χ*^(3)^.

## Conclusion

By extensively characterizing single- and few-layers flakes of 2H-WSe_2_ with photoluminescence, Raman spectroscopy and atomic force microscopy, we obtained the precise number of layers of each regions of the flake. We mapped the spatial emission of the second- and third-harmonic signals to study the layer dependence of the nonlinear response and quantified the corresponding susceptibilities. We obtained the values |*χ*^(3)^_s_| = (0.91 ± 0.07) × 10^−28^ m^3^ V^−2^ and |*χ*^(3)^_b_| = (1.16 ± 0.09) × 10^−19^ m^2^ V^−2^, which are comparable to the third-harmonic susceptibility of related semiconducting TMD and provide one step forward towards the complete characterization of the nonlinear optical properties of this family (MX_2_; M = W, Mo; X = S, Se). The reliability of these values is supported by the good agreement of our values for |*χ*^(2)^|, extracted from the simultaneously recorded second-harmonic signal, with previous reports for the same material. The strong nonlinear response of WSe_2_ in the infrared makes it a material suitable for applications in silicon photonics, all-optical switching and optical frequency conversion.

## Materials and Methods

### Sample fabrication and characterization

Flakes of WSe_2_ were obtained via micromechanical exfoliation from a single bulk crystal and transferred onto a fused silica substrate. To prevent degradation, the sample was partially covered with multi-layer hBN. The sample’s properties were characterized by photoluminescence, Raman spectroscopy and atomic force microscopy (for more details sample fabrication and characterization, see supporting information).

### Experimental setup

Nonlinear optical properties of WSe_2_ were investigated in a home-built multiphoton microscope setup. We used a half-waveplate to control the input linear polarization and a polarizer to analyze the harmonic signals. The pump laser was a 1545 nm, 200 fs, 80 MHz mode-locked fiber. A 100x objective lens focused the pump beam down to a spot size of 2 μm^2^. The step used in the sample displacement during spatial mapping was 0.5 µm (for more details on the experimental setup, see supporting information).

### Data availability

The datasets generated during and/or analyzed during the current study are available from the corresponding author on reasonable request.

## Electronic supplementary material

Supporting information
