# Supplementary material for: Characterization of the second- and third-harmonic optical susceptibilities of atomically thin tungsten diselenide
Source: Sci Rep. 2018 Jul 3;8:10035. doi: 10.1038/s41598-018-28374-1 (PMC6030073; doi:10.1038/s41598-018-28374-1)
Supplement: Supplementary file 1 — Supporting information [file 41598_2018_28374_MOESM1_ESM.docx]

Characterization of the second- and third-harmonic optical susceptibilities of atomically thin tungsten diselenide

Henrique G. Rosa^1,*^, Ho Yi Wei^1,2,3,*^, Ivan Verzhbitskiy^1,3^, Manuel J. F. L. Rodrigues^1,4^, Takashi Taniguchi^5^, Kenji Watanabe^5^, Goki Eda^1,3,6^, Vitor M. Pereira^1,3, †^, José C. V. Gomes^1,3, ‡^

^1^Centre for Advanced 2D Materials, National University of Singapore, 6 Science Drive 2, Singapore 117546

^2^NUS Graduate School for Integrative Sciences and Engineering (NGS), Centre for Life Sciences (CeLS), 28 Medical Drive, Singapore 117456

^3^Department of Physics, National University of Singapore, 2 Science Drive 3, Singapore 117551

^4^Center of Physics and Department of Physics, Universidade do Minho, 4710-057, Braga, Portugal

^5^National Institute for Materials Science, 1-1 Namiki, Tsukuba 305-0044, Japan

^6^Department of Chemistry, National University of Singapore, 3 Science Drive 3, Singapore 117543

*These authors contributed equally to this work

^†^Corresponding author email: vpereira@nus.edu.sg

^‡^Corresponding author email: phyvjc@nus.edu.sg

Key-words: Transition metal dichalcogenides, tungsten diselenide, second-harmonic generation, third-harmonic generation, nonlinear optical susceptibility.

# Supporting information

**Sample fabrication:** We fabricated the tungsten diselenide (WSe_2_) sample via micromechanical exfoliation from a bulk crystal and directly transferred onto glass substrate from the exfoliation tape. Then, multi-layer hexagonal boron nitride (hBN), approximately 16 nm thick, was transferred onto WSe_2_, partially covering the flake to protect it from environmental degradation^1,2^.

**Sample characterization:** We used photoluminescence (PL) microscopy and Raman spectroscopy to characterize sample’s properties and to identify monolayer regions, as shown in Figure S1.


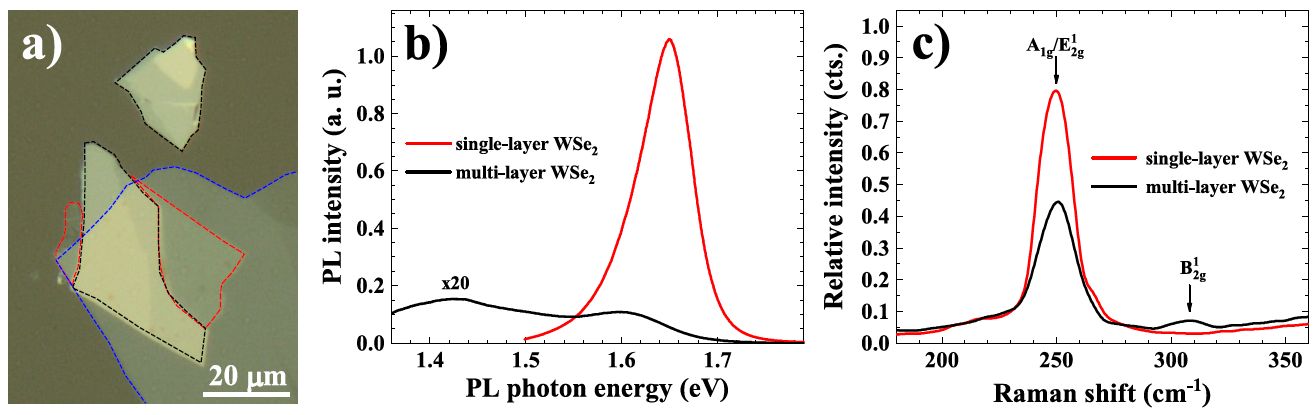


Figure S1. a) Optical image of WSe_2_/hBN structure - dashed red line: single-layer regions, dashed black line: multi-layer regions, dashed blue line: multi-layer hBN; b) photoluminescence and c) Raman spectra of single- and multi-layer regions.

From both PL and Raman spectra shown in Figure S1, one can distinguish between single- and multi-layer regions within the sample: single-layer region presents a prominent PL peak at 1.65 eV and degenerate A_1g_/E_2g_^1^ Raman peak at 249.6 cm^-13^. For all multi-layer regions, besides the flat and weak PL signal, the B_2g_^1^ Raman peak (characteristic of few-layers WSe_2_)^3^ is present. As expected, no PL or Raman peak associated with hBN were observed.

To unambiguously determine the precise number of layers (*N*) of each region, atomic force microscopy (AFM) was used to trace profile cuts from substrate onto each region of the sample. Figure S2 shows the AFM results.


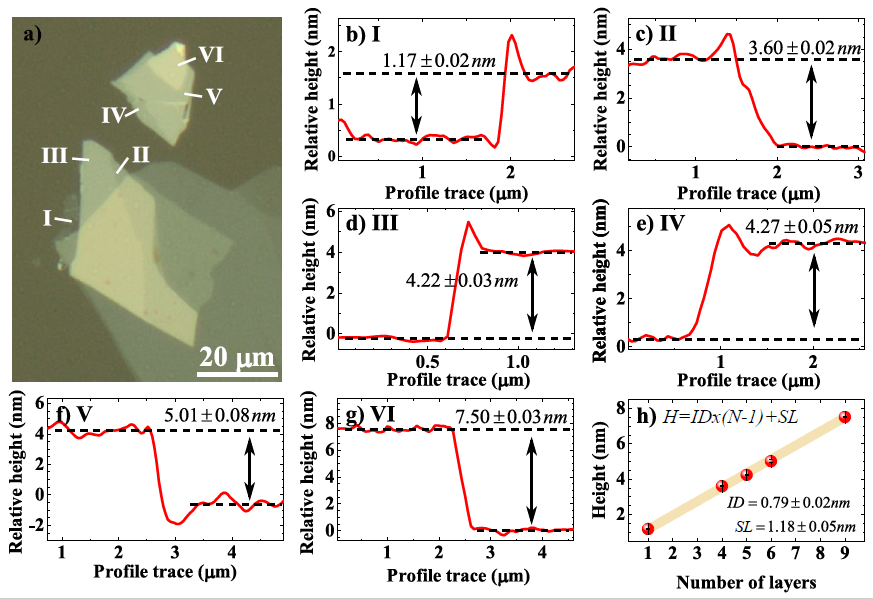


Figure S2. a) Sample optical image indicating the AFM profile traces location; b-g) profile traces I to VI; c) Measured height for each region, allowing the identification of the number of layers – ID: inter-layer distance, SL: single-layer thickness.

From PL and Raman results, trace I (Figure S2b) shows the thickness for the single-layer region to be ~1.17 nm, which agrees with previous published results^4–6^. The inter-layer separation distance is extracted from traces II – VI (Figure S2c-g), found to be in the 0.7-0.8 nm range. A linear fit (Figure S2h) yields single-layer thickness and inter-layer distance of 1.18 ± 0.05 nm and 0.79 ± 0.02 nm, respectively. To summarize, regions I to VI were shown to have 1, 4, 5, 5, 6 and 9 layers, respectively.

**Experimental setup:** Nonlinear optical properties of WSe_2_ were investigated in a home-built multiphoton microscope setup, as shown in Figure S3.


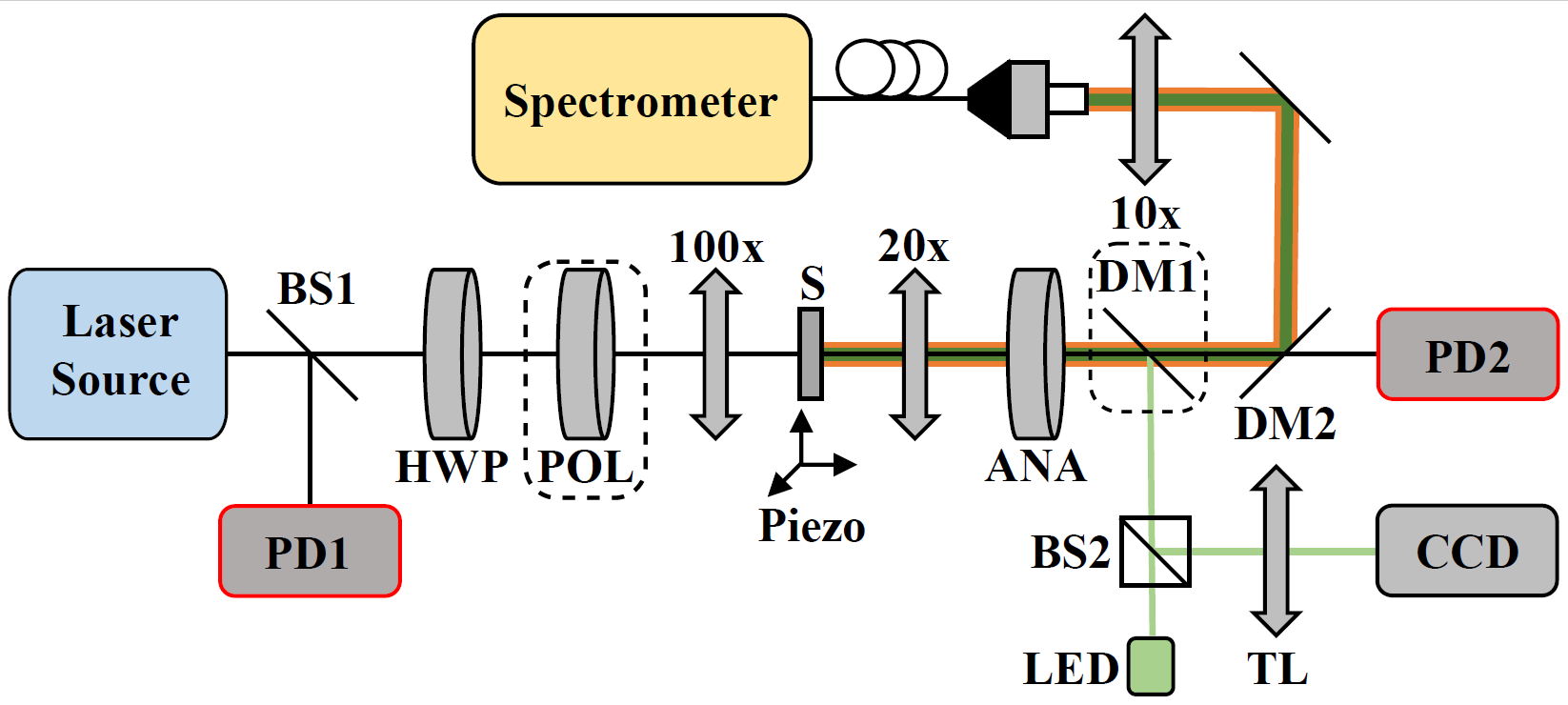


Figure S3. Multiphoton microscope experimental setup. BS: beam splitter; PD: photodetector; HWP: half-waveplate; POL: polarizer; 100x: focusing 100x objective lens; S: sample; 20x: imaging 20x objective lens; ANA: analyzer; DM: dichroic mirror; TL: tube lens; CCD: CCD imaging camera; 10x: coupling 10x objective lens.

The microscope setup has two parts: the first part is an optical microscope for locating and positioning the sample. For this purpose, DM1 is placed in the setup, allowing sample’s illumination and back-reflected light collection. A piezoelectric stage is docked on a mechanical translational stage to control sample’s position precisely. The sample is mounted on the setup such that the WSe_2­_ flake faces the 20x objective.

The second part is the multiphoton microscope for SHG and THG measurements. In this experiment, a 1550 nm, 200 fs, 80 MHz mode-locked fiber laser was used as pump beam. The 100x objective lens focused the pump beam down to a spot size of 2 μm^2^. The average power of the pump, at the sample, was 0.65 mW. The sample was mounted on the setup such that the WSe_2­_ flake faces the 20x objective. After the sample, pump and harmonic beams were collimated by a 20x objective lens, then separated by DM2. The pump was recorded by a reference photodetector (PD2) while harmonic signals were coupled to an optical fiber by a 10x objective lens and detected by a TEC-CCD spectrometer (Avantes HERO) with 10 s acquisition time. All characterization and experiments were performed at room temperature.

As can be seen from Figure S1, the optical bandgap energy of the single-layer flake is 1.65 eV, which places both second- and third-harmonic signals off resonance, therefore no signal enhancement caused by excitonic effect was observed.

The calibration factors for the actual power reading by the spectrometer were determined by simultaneously measuring the power of a reference laser beam in a powermeter and in the spectrometer (with calibrated attenuators). The calibration factor for 775 nm (SHG) is
0.275 fW counts^-1^ and for 516 nm (THG) is 0.512 fW counts^-1^, accounting for the direct relation of generated harmonics power at the sample and the spectrometer reading.

**Transmitted pump power mapping:** We recorded simultaneously the transmitted pump power for each position of the harmonic mappings, therefore being able to also map the WSe_2_ sample in transmittance at the fundamental wavelength. The pump beam map is shown in Figure S4. From this map we can observe that different regions of the sample, depending upon the WSe_2_-hBN stacking structure, have different transmitted pump powers. This can be attributed to the different stacking at each region or even two-photon absorption. We did not investigate further for the latter effect as it is not the focus of this report.


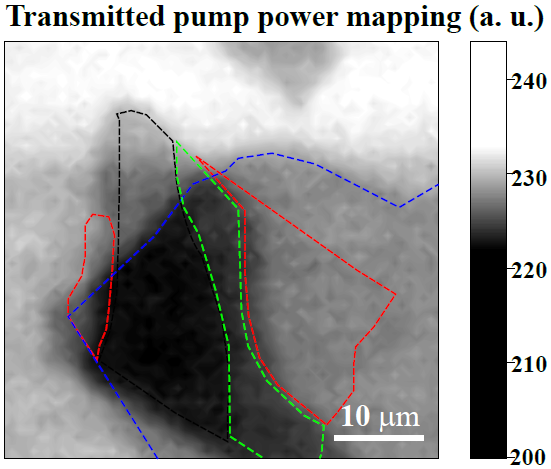


Figure S4. Pump beam transmittance mapping.

**References:**

1. Ahn, S. *et al.* Prevention of Transition Metal Dichalcogenide Photodegradation by Encapsulation with h-BN Layers. *ACS Nano* **10,** 8973–8979 (2016).

2. Lee, G.-H. *et al.* Highly Stable, Dual-Gated MoS_2_ Transistors Encapsulated by Hexagonal Boron Nitride with Gate-Controllable Contact, Resistance, and Threshold Voltage. *ACS Nano* **9,** 7019–7026 (2015).

3. Tonndorf, P. *et al.* Photoluminescence emission and Raman response of monolayer MoS_2_, MoSe_2_, and WSe_2_. *Opt. Express* **21,** 4908–4916 (2013).

4. Pawbake, A. S., Pawar, M. S., Jadkar, S. R. & Late, D. J. Large area chemical vapor deposition of monolayer transition metal dichalcogenides and their temperature dependent Raman spectroscopy studies. *Nanoscale* **8,** 3008–3018 (2016).

5. Li, H. *et al.* Rapid and reliable thickness identification of two-dimensional nanosheets using optical microscopy. *ACS Nano* **7,** 10344–10353 (2013).

6. Resta, G. V. *et al.* Polarity control in WSe_2_ double-gate transistors. *Sci. Rep.* **6,** 29448 (2016).
